# Fulminant Shigellosis in a HIV Patient 

**DOI:** 10.1155/2015/128104

**Published:** 2015-03-04

**Authors:** Siang Mei Sally Ooi

**Affiliations:** Liverpool Hospital, Elizabeth Street, Liverpool, NSW 2170, Australia

## Abstract

Infectious enterocolitis caused by *shigella* is usually self-limiting and seldom requires antibiotics treatment. It is uncommon to develop fulminant shigellosis requiring surgery. We report a rare case of fulminant shigellosis in a HIV patient with recurring infection which could not be managed with intravenous antibiotics. CT reviewed extensive colonic wall thickening and stranding with evidence of pneumatosis coli. The patient eventually required a Hartmann procedure. Although fulminant shigellosis is uncommon, thorough assessment and vigilant management are warranted in immunosuppressed patient.

## 1. Introduction

Infectious enterocolitis is usually self-limiting in the healthy immunocompetent population. In the context of HIV infection, gastrointestinal symptoms such as anorexia, weight loss, abdominal pain, and diarrhoea are frequent and nonspecific. Infectious agents such as Clostridia,* Salmonella*,* Shigella*, and* Campylobacter* may resist treatment. HIV enteropathy may cause ileal dysfunction, altered bowel motility, and bacterial overgrowth. Opportunistic organisms such as* Cytomegalovirus*,* Candida*,* Cryptococcus*, and* Strongyloides* may yield disease. Here, we present a patient with HIV and fulminant shigellosis.

## 2. Case Report

Patient A is a 44-year-old man with a background of developmental delay, previous right inguinal herniorrhaphy, sensorineural hearing loss, and HIV. His routine medications include antibiotic prophylaxis and highly active antiretroviral therapy. Patient A was admitted with recurrent shigellosis despite oral ciprofloxacin. He had suffered a month of vomiting, foul-smelling watery diarrhoea, 12 kg weight loss, low-grade fever, and generalised abdominal pain. Stool cultures had confirmed* Shigella flexneri*, for which the infectious disease team had placed him on ciprofloxacin. On examination, he had a blanching maculopapular rash over his trunk and limbs. His abdomen was distended, with tenderness and evidence of peritonism in the left lower quadrant. Abdominal CT scan demonstrated pancolonic wall thickening, extensive pericolic stranding, pneumatosis coli of the descending colon, and extraluminal gas locules.

**Figure 1:**
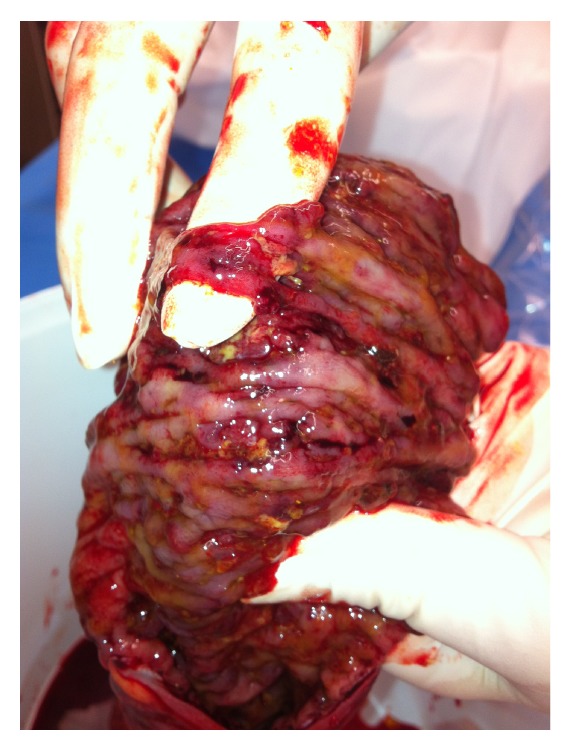
Extensive ulceration affecting the descending and sigmoid colon.

**Figure 2:**
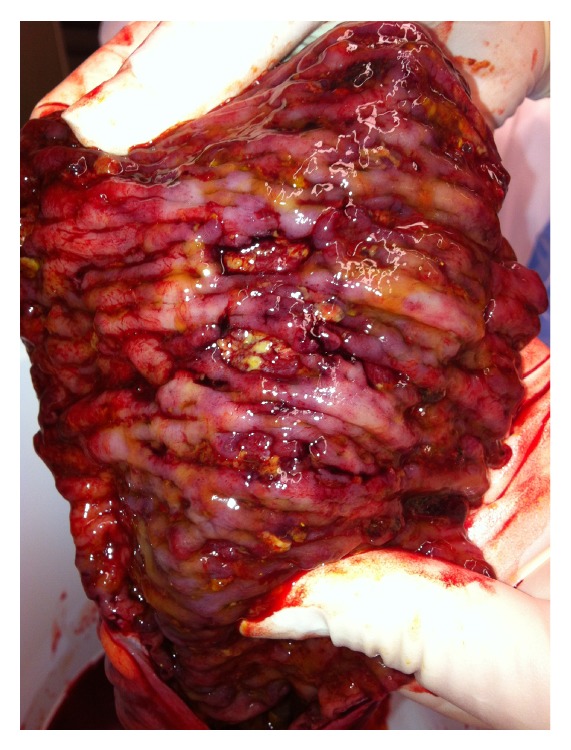
Postoperative specimen.

**Figure 3:**
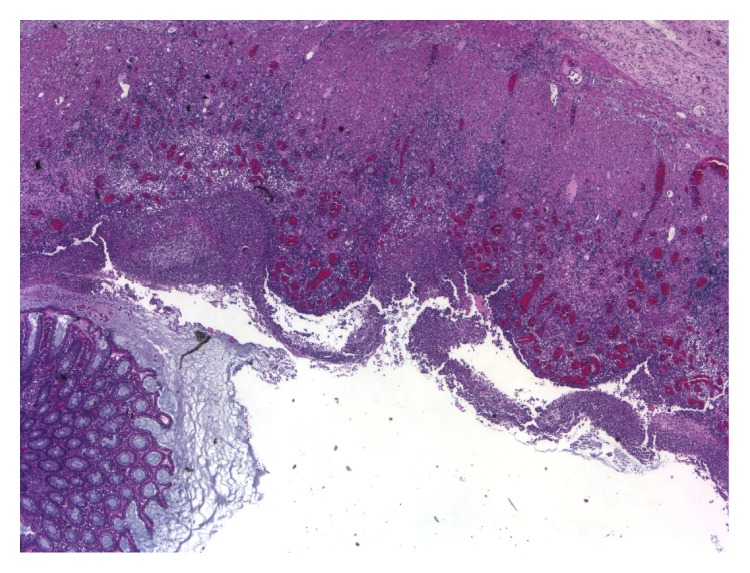
Ulceration of the colonic mucosa, low power view.

At laparotomy, there were extensive oedema and inflammation affecting the splenic flexure and distal transverse and sigmoid colon. The descending colon was friable with palpable crepitus and extensive ulceration (Figures 1 and 2). A Hartmann procedure was performed. Histopathology demonstrated extensive ulceration into the deep submucosa and muscularis propria, transmural oedema, pericolic fibrosis, venous congestion, pseudopolyp formation, and an overlying thick pseudomembrane of fibrin, mucin, and acute inflammatory cells (Figure 3).

Postoperatively, patient A was slow to reestablish oral intake and required parenteral nutrition. He was eventually discharged to a group home with a plan for colonoscopy prior to reversal of ileostomy.

## 3. Discussion

*Shigella* is nonmotile, facultatively anaerobic, indole-positive, urea- and oxidase-negative gram-negative rods that ferment glucose [1]. There are four members of the family Enterobacteriaceae, genus* Shigella*—*dysenteriae*,* flexneri*,* boydii*, and* sonnei*—which can be cultured with routine laboratory techniques on MacConkey or eosin methylene blue media [2].

*Shigella* invades colonic enterocytes and specialised epithelial M cells overlying lymphoid follicles and induces an intense inflammatory response, leading to the death of epithelial and immune cells, colonic mucosal ulceration, and abscesses [3].* Shigella* strains elaborate chromosomally encoded ShET1 enterotoxin, virulence plasmid-encoded ShET2 enterotoxin, and Shiga toxin. They are less susceptible to acid than other bacteria; as few as 10 to 100 organisms are capable of causing bloody mucoid diarrhoea, abdominal cramps, and high fevers [4].

*Shigella* gastroenteritis is usually self-limiting; however, the spectrum of disease severity varies depending on the serogroup—mild disease with* Shigella sonnei* to fulminant dysentery with* Shigella dysenteriae 1* or* flexneri* [1]. The incubation period ranges from one to seven days [5].

Transmission occurs via direct person-to-person contact and faecal-oral spread, particularly in day care centres or custodial institutions, and less commonly through contaminated food [5]. In a population-based case-control study, HIV infection, direct oral-anal contact, and men who have sex with men were each associated with an odds ratio for shigellosis of 7.5 to 8.2 [6].

Intestinal complications are rare and include

1. severe inflammation of the rectosigmoid leading to rectal prolapse, particularly in infants and young children;
2. pancolitis, fulminant colitis and toxic megacolon, and the sequelae of intense inflammation in the setting of* S. dysenteriae 1*; in Bangladesh, up to 3% of patients with shigellosis present with toxic megacolon [7];
3. intestinal obstruction occurring in up to 2.5%, usually related to* S. dysenteriae 1* [7];
4. colonic perforation, principally in infants or severely malnourished patients, which is associated with* S. dysenteriae 1* or* S. flexneri* [8];
5. systemic manifestations including bacteremia, shock, hyponatraemia, and leukemoid reaction, particularly in children younger than five years of age [9];
6. neurological manifestations ranging from encephalopathy and cerebral oedema to obtundation, coma, and Ekiri syndrome [7]; seizures were once attributed to circulating shiga toxin but may be linked to other enterotoxins [10];
7. possibility of protein-losing enteropathy, haemolytic-uraemic syndrome, and reactive arthritis or Reiter's syndrome occurrence [7].

Management is dependent on disease severity. Antibiotics are not universally essential as infection clears spontaneously in most cases [11]. However, in patients like A, the degree of immunodeficiency, in addition to the organism's innate virulence, determines the degree of mucosal damage. Empiric therapy with fluoroquinolones is generally warranted in the elderly, malnourished, those with HIV, food handlers, health care workers and childcare workers while waiting for cultures and antibiotics sensitivities.

Fulminant shigellosis requiring resection is uncommon in Australia. Patients with immunocompromised states need to undergo careful assessment and close surveillance during treatment for serogroups that would otherwise run a self-limiting course.
